# A robust self-supervised approach for fine-grained crack detection in concrete structures

**DOI:** 10.1038/s41598-024-63575-x

**Published:** 2024-06-02

**Authors:** Muhammad Sohaib, Md Junayed Hasan, Mohd Asif Shah, Zhonglong Zheng

**Affiliations:** 1 School of Computer Science and Technology, Zhejiang Normal University, Jinhua, 321004 China; 2 Zhejiang Institute of Photoelectronics and Zhejiang Institute for Advanced Light Source, Zhejiang Normal University, Jinhua, 321004 China; 3 National Subsea Centre, Robert Gordon University, Aberdeen, AB21 0BH UK; 4 Department of Economics, Kardan University, Parwane Du, Kabul, 1001 Afghanistan; 5 Division of Research and Development, Lovely Professional University, Phagwara, Punjab 144001, India

**Keywords:** Concrete cracks detection, Curriculum learning, Gaussian adaptive weights, Pseudo-labeling, Structural health monitoring, Self-supervised YOLO, Computational science, Computer science, Information technology, Scientific data, Civil engineering, Energy infrastructure

## Abstract

This work addresses a critical issue: the deterioration of concrete structures due to fine-grained cracks, which compromises their strength and longevity. To tackle this problem, experts have turned to computer vision (CV) based automated strategies, incorporating object detection and image segmentation techniques. Recent efforts have integrated complex techniques such as deep convolutional neural networks (DCNNs) and transformers for this task. However, these techniques encounter challenges in localizing fine-grained cracks. This paper presents a self-supervised 'you only look once' (SS-YOLO) approach that utilizes a YOLOv8 model. The novel methodology amalgamates different attention approaches and pseudo-labeling techniques, effectively addressing challenges in fine-grained crack detection and segmentation in concrete structures. It utilizes convolution block attention (CBAM) and Gaussian adaptive weight distribution multi-head self-attention (GAWD-MHSA) modules to accurately identify and segment fine-grained cracks in concrete buildings. Additionally, the assimilation of curriculum learning-based self-supervised pseudo-labeling (CL-SSPL) enhances the model's ability when applied to limited-size data. The efficacy and viability of the proposed approach are demonstrated through experimentation, results, and ablation analysis. Experimental results indicate a mean average precision (mAP) of at least 90.01%, an F1 score of 87%, and an intersection over union threshold greater than 85%. It is evident from the results that the proposed method yielded at least 2.62% and 4.40% improvement in mAP and F1 values, respectively, when tested on three diverse datasets. Moreover, the inference time taken per image is 2 ms less than that of the compared methods.

## Introduction

Deterioration of concrete structures due to cracks can happen due to different factors including drying shrinkage, stress, chemical reaction, corrosion, substandard construction practices. The strength and durability of concrete structures are compromised due to occurrence of cracks^1^. Therefore, it is inevitable to properly identify and locate the cracks during routine inspections to avoid losses and catastrophes^2^. Previously, the evaluation of the concrete structures used to be performed manually. However, the manual inspection has its own cons, for instance, reliance on the expertise of the inspector, tedious, and substantial time commitment. Moreover, manual inspection is coupled with inherent safety risks. Over the years, researchers have implemented various automated techniques to assess damage in concrete structures using computer vision (CV) techniques^3^.

These techniques can be classified into two classes, i.e., object detection and image segmentation^4^. In object detection techniques cracks are identified and classified on in concrete structures using bounding boxes. Whereas, image segmentation techniques are used to rigorously extract the mask representing the shape and pattern of the cracks. The information obtained by the segmented cracks is beneficial for engineers to evaluate the safety of a structure^5^. Initially, predominantly image processing techniques (IPTs) were used in crack segmentation research. However, these IPT-based techniques necessitate a comprehensive feature engineering by experts to efficiently identify cracks with complex shapes and improve the generalization ability of the methods^6^.

To mitigate these issues, machine learning and deep learning approaches have been incorporated in intelligent crack detection and segmentation techniques^7,8^. The emergence of advance architectures, such as deep convolutional neural networks (DCNN)^9^, U-shape networks (UNets)^10^, DeepLab^11^, W-Segnet^12^ and you only look once (YOLO)^13^ models, have enormous contribution in the increasing popularity of deep learning-based image segmentation. Several improvements have been incorporated into these networks to further improve their performance. One of the improvements is to use sophisticated pre-trained models like ResNet34^14,15^ and EfficientNet^16^ to enhance the detection and segmentation accuracy. Similarly, to improve the performance of a network inclusion of powerful fusion modules like the multi-scale fusion^17^ and the skip-squeeze-and-excitation^18^ modules can also be valuable. Likewise, residual connections^19^ and attention mechanisms^20^ can aid in exploring vital, contextual information. In addition, inclusion of pre-processing^21^ and post-processing^22^ modules can augment the performance of a model. Recently, numerous experiments have been enacted to elevate the crack detection and segmentation efficacy in realistic and obscure scenarios. Xu et al.^23^ proposed an effectual mechanism for classifying cracks in steel box girders encompassing handwritten inscriptions and welds based on a fused convolutional neural network (CNN). To alleviate the impact of obscure backgrounds this approach examined small image blocks. Nevertheless, additional effort is required for improving the detection accuracy as it lacks the global perception of the cracks. Similarly, Zhong et al.^24^ developed an algorithm for generating synthetic images of grooved concrete pavement cracks using a deeper generative adversarial network. Additionally, they utilized U-Net and W-Segnet for achieving pixel-level crack detection. Their findings indicated that both W-Segnet and U-Net demonstrated improved pixel-level segmentation results when trained on the synthesized data. Moreover, Choi et al.^25^ introduced semantic damage detection network (SDDNet) by incorporating various additional modules into the CNN. The proposed model alleviated the impact of intricate backgrounds and crack-like properties. In addition, Zhong proposed a pavement distress detection network and applied it to the images captured through unmanned aerial vehicle. The designed approach worked better than R-CNN, U-Net, and W-segnet^26^. The aforementioned models exhibit promising crack detection results under certain circumstances, but the incorporation of additional efforts is essential for more reliable crack detection in broader contexts^27^.

These segmentation models had encoder-decoder architectures with CNN as a backbone network^28^. It is difficult for these models to accurately model the global features of cracks under complex and practical detection situations^29,30^. Moreover, the encoder which implements convolution operations in a sequential manner could lead to forfeiting the localization and contextual information of a target during the down sampling process. Furthermore, the decoder relies on the mapping of higher-order features, often ignoring the feature mappings of lower-order features that could provide detailed spatial information^31^. An increment in the receptive field of a network using deeper or atrous convolution can mitigated this issue. Ali et al. proposed a mechanism to expand the receptive field and retrieve global characteristics by utilizing different dilation ratios^32^. However, this method can lead to certain issues including a loss of local information, challenges with feature reuse during training, and a reduced spatial resolution of the images. Thus, there is a gradual amelioration in the capability of the model to explore global features^33^. Alternatively, attention mechanism is used to enhance the capability of a network to explore global features. Nonetheless, the subtleties of nearby object may distract or confuse during crack segmentation, making it more challenging to build a global pattern at the object level. Additionally, convoluting local features and incorporation of standard attention mechanism for global features may cause ambiguity during training. It can hinder the network from leveraging the two aforementioned methods for subtle information exploration at different scales^34^. Therefore, crack segmentation requires more effective mechanism for representing global contextual information and extract details regarding low-level features.

One of the most innovative and compelling approaches to resolve this dilemma is the incorporation of a transformer. Transformers are deep learning networks that incorporate both self-attention and embedding which makes them different from CNNs^35^. In natural language processing (NLP), transformers are known for long-range modelling and global information extraction^36^. Likewise, transformers are frequently applied in computer vision applications, for instance, images classification, target identification, and semantic segmentation. The first computer vision application of transformer network was introduced by Vision Transformer (ViT)^37^. Recent research indicates that transformer models can excel in crack detection and segmentation as compare to CNN models. Yan et al.^38^ proposed an end-to-end crack detection network based on transformer, which excelled the performance of traditional deep networks. Analogously, a vision transformer (Vit)-based approach is introduced by Shamsabadi et al.^39^ has been utilized as an encoder-decoder manner to generate better crack detection results. Moreover, Guo et al. proposed SegFormer and swin-transformer based approach, which demonstrated higher performance in cracks segmentation task^40^. In their computationally efficient approach, SegFormer was used as a multilayer perceptual decoder and the swin-transformer was used as encoder. Various hybrid approaches, integrating CNN-transformer for segmenting cracks have been proposed to address the issues transformer models encounter when acquiring local information. In crack detection and segmentation improved transformer based models such as SegCrack^41^ and CGTr-Net^42^ have shown encouraging results.

Despite the availability of models incorporating CNN as backbone in transformer networks, more sophisticated approaches are still needed to solve the challenges of crack segmentation. Whereas a transformer receiving inputs in a sequence may replicate the global context of each phase, its capability to capture local details is restricted^43^. Additionally, the process of upsampling is uncapable of restoring low-resolution feature maps, as sole reliance on transformer is insufficient to provide accurate localization of fine-grained cracks. Moreover, contrary to the available crack detection and segmentation datasets, transformer based model necessitates a substantial dataset to effectively used its self-attention mechanism^32^. Furthermore, practical scenario necessitates crack detection and segmentation models with low latency and high inference speed^44^.

In several studies, different variants of You only Look once (YOLO) network have been utilized as a remedy for the real-time crack detection and segmentation problem due to their high inference speed and low latency. Qiu et al.^45^ explored that ResNet based YOLOv3 and YOLOv4-tine are suitable for unmanned aerial vehicle (UAV) based real-time detection of cracks. Likewise, maintaining the original dimension-YOLO (MOD-YOLO) based crack detection techniques is presented which enhanced accuracy and generalizability for the task. Although, these algorithms provide satisfactory results under certain conditions, yet, there are a few issues associate with these approaches. The first and foremost issue is to improve the performance of a model in the presence of limited-size dataset, as it is the case with crack detection and segmentation datasets in concrete structures. Secondly, the presence of fine-grained cracks, i.e., cracks with small, narrow, and subtle nature poses a challenge for automated detection process and can adversely affect the performance of a designed model. Lastly, the ability to detect cracks of these algorithms deteriorates with varying complex backgrounds implying the low generalizability of algorithms.

An effectual approach is proposed in this work to infer and segment fine-grained cracks. The aim is to improve the performance of the model with limited-size dataset having varying complex backgrounds with strong generalization to diverse scenarios. The proposed approach uses a YOLOv8 model integrating concepts of self-supervised labeling and attention mechanism to design self-supervised you only look once (SS-YOLO) model. It incorporates convolution block attention module (CBAM) and Gaussian adaptive weight distribution multi-head self-attention module (GAWD-MHSA). The inclusion of attention modules empowers the model to efficaciously attain subtle information and variations coupled with fine-grained cracks by prioritizing relevant information. In addition, it uses curriculum learning based self-supervised pseudo-labeling (CL-SSPL) to improve the learning ability of the network on limited-size dataset ensuring high generalizability. The prime contributions of this paper are presented as follow:

1. The utilization of pseudo-labeling approach in the presence of a limited-size dataset to elevate the generalizability of the proposed model. It augments the training data by leveraging predictions on unlabeled dataset. As a result, representation learning capability of the model improves and address data imbalance. The CL-SSPL also serves as a method of regularization, and aids in the adaptation to varying data distributions.
2. Integration of CBAM, and GAWD-MHSA modules to explore meaningful insights and variations associated with these cracks in a better way. These attention modules empower the proposed model to highlight pertinent details, making the model more resilient in coping intricate backgrounds and salient information.

The next section presents the overview of YOLOv8 segmentation network followed by the proposed the description of self-supervised adaptive muti-attention YOLOv8 model. The details of experimental setup are given in “Experimental setup”. Section “Results and analysis” illustrates the analysis of the key results and “Discussion” is regarding the discussion on this work. Lastly, the paper is concluded in “Conclusion”.

## YOLOv8 segmentation network

**Figure 1:**
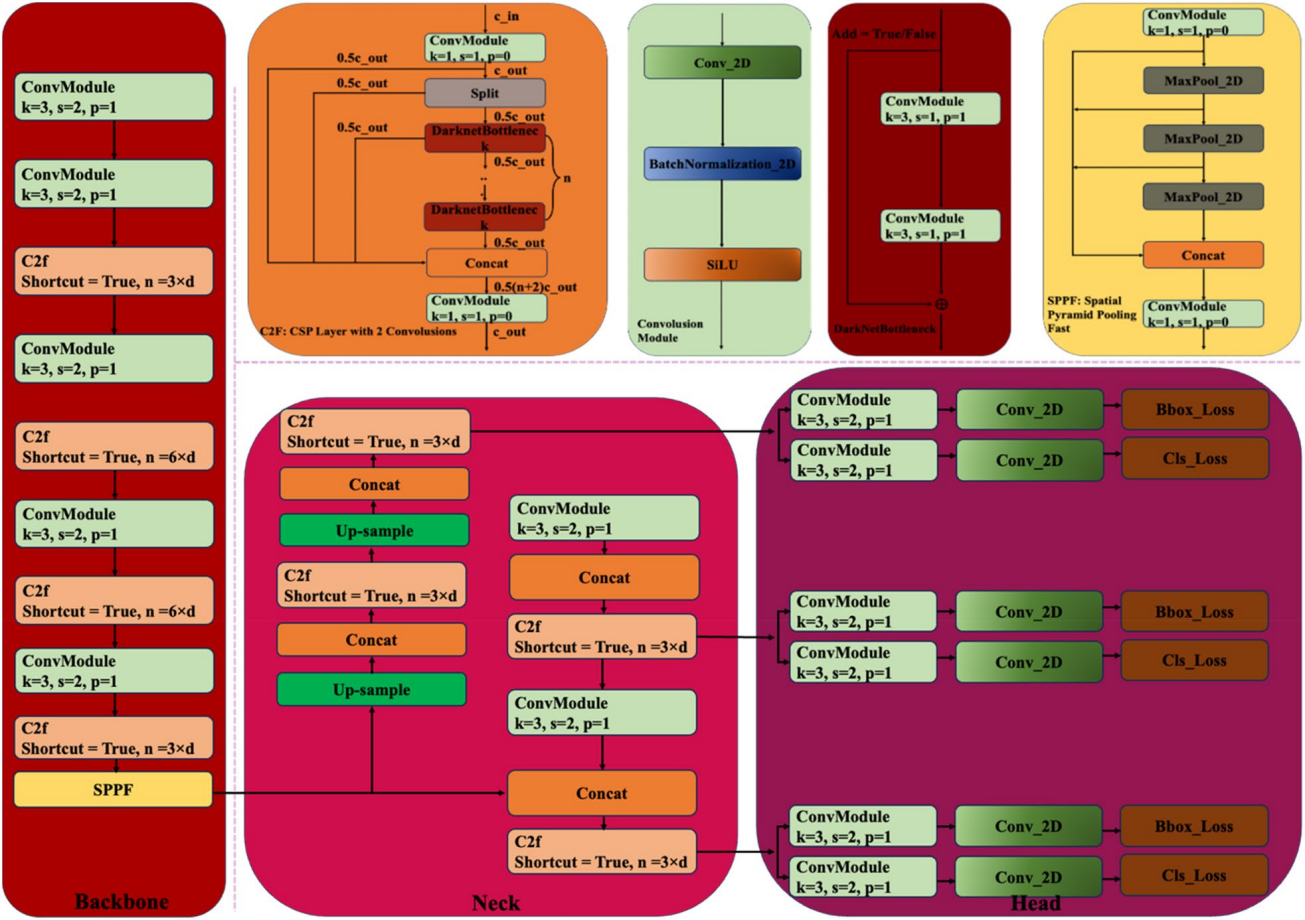
The illustration of the basic architecture of YOLOv8.

YOLOv8 is a member of the family of you only look once (YOLO) networks introduced by Ultralytics^46^. It is the same organization that introduced YOLOv5. There are five versions of YOLOv8, namely YOLOv8n (nano-version), YOLOv8s (small-version), YOLOv8m (medium-version), YOLOv8l (large-version) and YOLOv8x (extra-large-version). These variants are suitable for various computer vision tasks including pose estimation, object detection, segmentation, and classification. The main features in the architecture of YOLOv8 presented in Fig. 1 are discussed in the following subsections. In this study, to avoid computational overhead, we considered the smaller version of YOLOv8, namely YOLOv8s.

### Backbone network

In YOLOv8, inputs undergo five-phase down sampling process through a backbone network based on a customized CSPDarknet53, producing five distinct feature scales. One of the key modifications introduced in YOLOv8 is the replacement of traditional Cross-Stage Partial (CSP) bottleneck modules with a more efficient architecture known as the C2f module, which consists of two convolutional operations. This modification aims to improve processing speed while maintaining effectiveness. The C2f module integrates features extracted by the backbone CSPDarknet53 network with Spatial Pyramid Pooling Fast (SPPF), facilitating robust object detection by combining semantic and multiscale information from the backbone network and Feature Pyramid Network (FPN), respectively. With two parallel branches for gradient flow, the C2f module enhances robustness and computational efficiency. Leveraging fewer convolutional operations and a refined information flow mechanism, the resulting network is lightweight with reduced computational complexity. The use of the Sigmoid-weighted Linear Unit (SiLU) aids in acquiring outputs from the C2f module. Additionally, constant-size feature maps produced by SPPF expedite the pooling process and enable adjustable output dimensions. The architecture also includes a decoupled head structure to independently compute classification, objectiveness, and regression losses^47^.

### Neck module

The neck module in YOLOv8 incorporates the Path Aggregation Network as well as Feature Pyramid Network (PAN-FPN), inspired by the PANet architecture. In contrast to its predecessors, namely YOLOv5 and YOLOv6, the convolution operation proceeding up sampling proceeding is excluded in YOLOv8, making the network more streamlined and lightweight model. It also creates a diverse and comprehensive feature pool by concatenating semantic information from shallow as well as deep levels.

### Head module

The architecture of the head module of YOLOv8 works in a decoupled fashion. It contains distinct routes for classification and bounding box regression. As it adopts an anchor-free approach, the head module is able to efficiently identifying the positive and negative samples. A dynamic assignment approach called Task-Aligned Assigner (TAA) is used to assign samples during the detection process, enhancing overall accuracy.

### Calculation of the loss

The decoupled head structure is efficient but has a tendency of potential misalignment when it performs localization and classification operations simultaneously. The TAA helps the head detection module to navigate through this problem by helping the model in distinguishing between positive and negative samples. It measures the accuracy of predicted bounding box by combining the classification score (CS) with the Intersection over Union (IoU) score. The estimated alignment score helps in the selection of top k number of positive samples and calculates a classification via Binary Cross-Entropy (BCE), as well as, regression loss with the help of Complete Intersection over Union (CIoU)^48^ and Distributional Focal Loss (DFL)^49^. BCE quantifies the difference between binary predictions and true labels, whereas, CIoU measure the difference between predicted bounding box and ground truth in terms of center point and aspect ratio. Moreover, DFL helps in the optimization of the distribution of the predicted bounding box boundaries by highlighting misclassified false negative samples. The mathematical formulation of the $CIoU$ and $DFL$ is given as follows^48,49^.

1 $$\begin{aligned} & Loss - CIoU = 1 - IoU + \frac{{D^{2} \left( {bx,bx^{{GT}} } \right)}}{{\left( {MB_{w} } \right)^{2} + \left( {MB_{h} } \right)^{2} }} + \frac{4}{{\pi ^{2} }}\left( {{\text{tan}}^{{ - 1}} \frac{{w^{{GT}} }}{{h^{{GT}} }} - {\text{tan}}^{{ - 1}} \frac{w}{h}} \right) \\ & DFL\left( {P_{r} \left( {l_{i} } \right),P_{r} \left( {l_{{i + 1}} } \right)} \right) = \left( {\left( {l_{{i + 1}} - l} \right){\text{log}}P_{r} \left( {L_{i} } \right) + \left( {l - l_{i} } \right){\text{log}}P_{r} (l_{{i + 1}} )} \right) \\ \end{aligned}$$

where, $IoU$ is the shortform of intersection over union. It denotes the ratio of intersection between the predicted and actual bounding boxes. Furthermore, the Euclidean distance between the predicted and the actual bounding boxes is represented by $D\left(bx,{bx}^{GT}\right)$. The height and width of the predicted box are denoted by ${p}^{h}$ and ${p}^{w}$, whereas, the height of the actual bonding box is denoted by ${h}^{GT}$ and width by ${w}^{GT}$. Similarly, ${MB}_{w}$ and ${MB}_{h}$ identify the width and height of the minimum box that encloses the prediction and true boxes.

In addition to the $CIoU$ YOlOV8 also takes advantage of the $DFL$ to rapidly regress over the values near a label $l$ by enlarging the probabilities for ${l}_{i}$ and ${l}_{i+1}$. As a result, it increases the optimization efficiency of the model by predicting the label with high confidence. In this equation, ${P}_{r}\left({l}_{i}\right)$ and ${P}_{r}\left({l}_{i+1}\right)$ represent the distribution of labels ${l}_{i}$ and ${l}_{i+1}$.

The YOLOv8 model has a limitation in that it relies solely on labeled data for training, which can be a hindrance in situations where labeled data is scarce or insufficient. Since the model depends exclusively on annotated data, it may struggle to generalize well to diverse and unforeseen environments, leading to difficulties in capturing robust features and patterns. Additionally, the process of obtaining a large amount of labeled data can be slow and expensive, limiting the scalability and real-world applicability of YOLOv8. Furthermore, YOLOv8 lacks the adaptability and flexibility required to handle unlabeled data, which can pose challenges in complex situations and potentially reduce its performance. These issues can be addressed by introducing a self-supervised pseudo-labeling approach during the training phase of the network.

## The self-supervised YOLO (SS-YOLO) segmentation network

**Figure 2:**
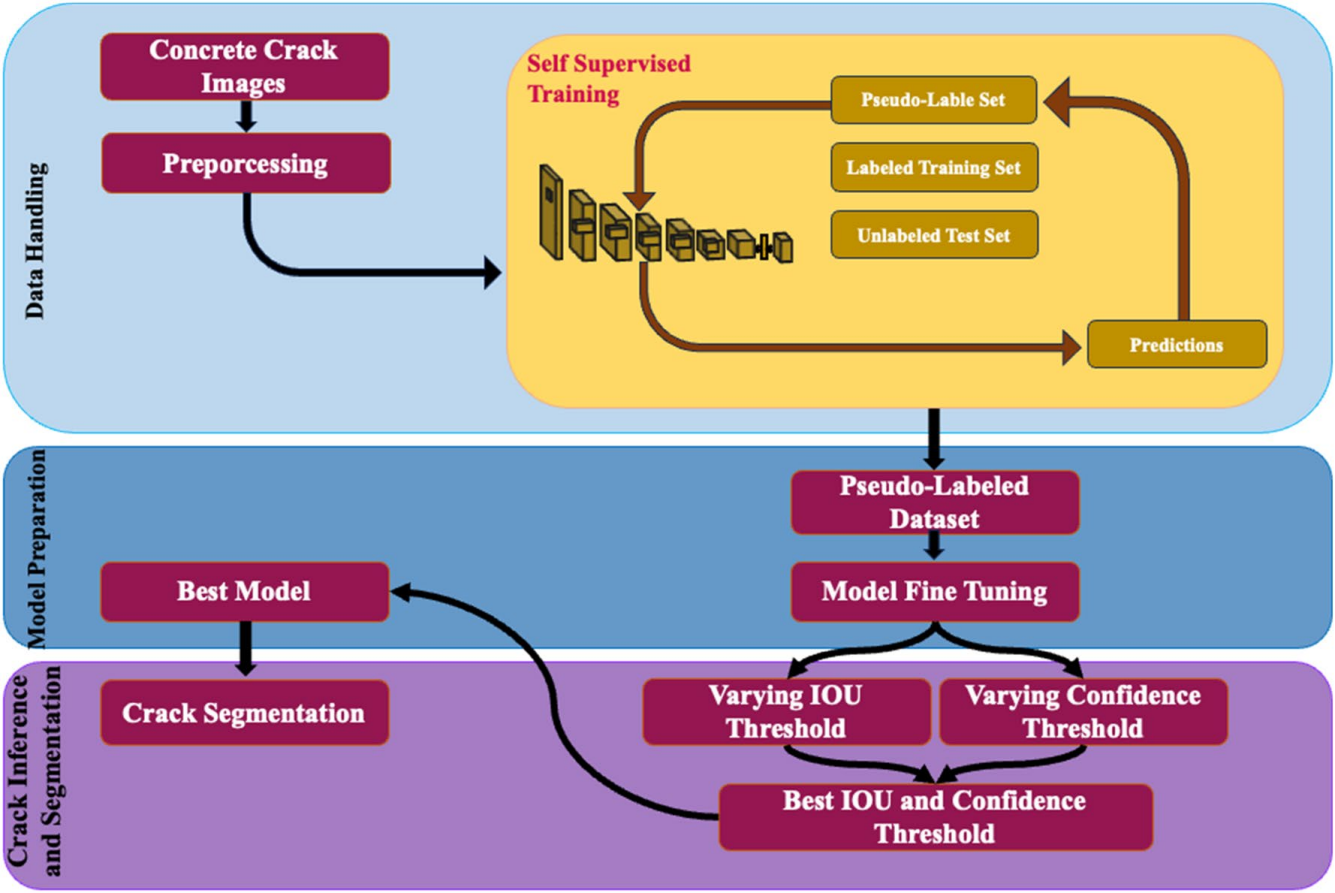
The Proposed self-supervised approach for the segmentation of fine-grained cracks.

**Figure 3:**
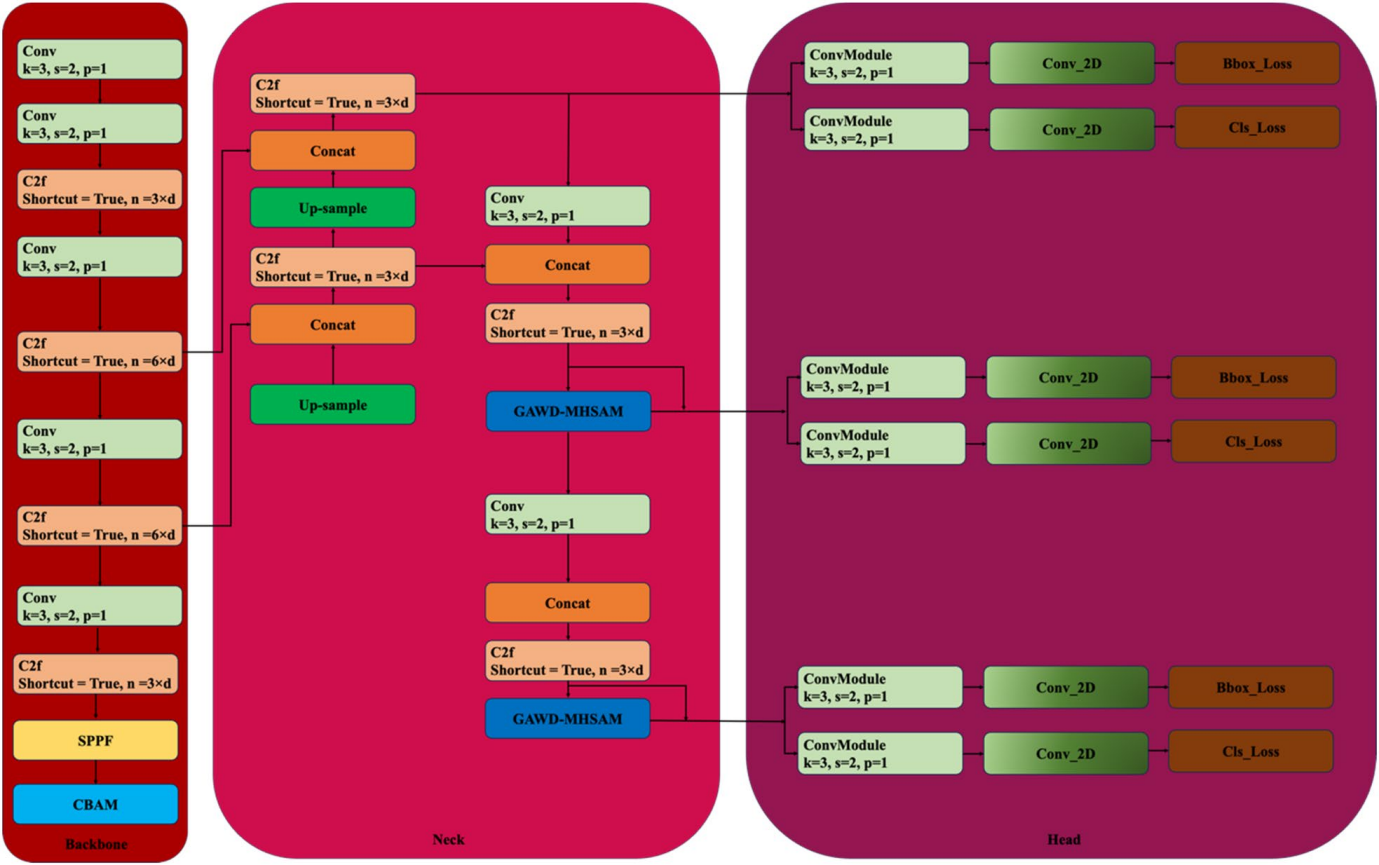
The illustration of the proposed SS-YOLO architecture.

The approach used for the segmentation of fine-grained cracks namely self-supervised YOLO (SS-YOLO) is presented in Fig. 2. The structure of the proposed SS-YOLO that builds upon the YOLOv8 architecture is illustrated in Fig. 3. The model introduces a self-supervised pseudo-labeling training mechanism for proficient representation learning through the network. It also utilizes innovative techniques to enhance its capability in processing features from different layers, with a particular focus on discriminating crack and background details. This improvement involves the assimilation of path aggregation network with GAWD-MHSAM. The incorporation of CBAM attention module in the backbone refines the its output features map to accentuate the attention on the regions associated with cracks. These augmentations collectively form the foundational framework of the SS-YOLO network.

### Self-supervised *pseudo*-labeling

**Figure 4:**
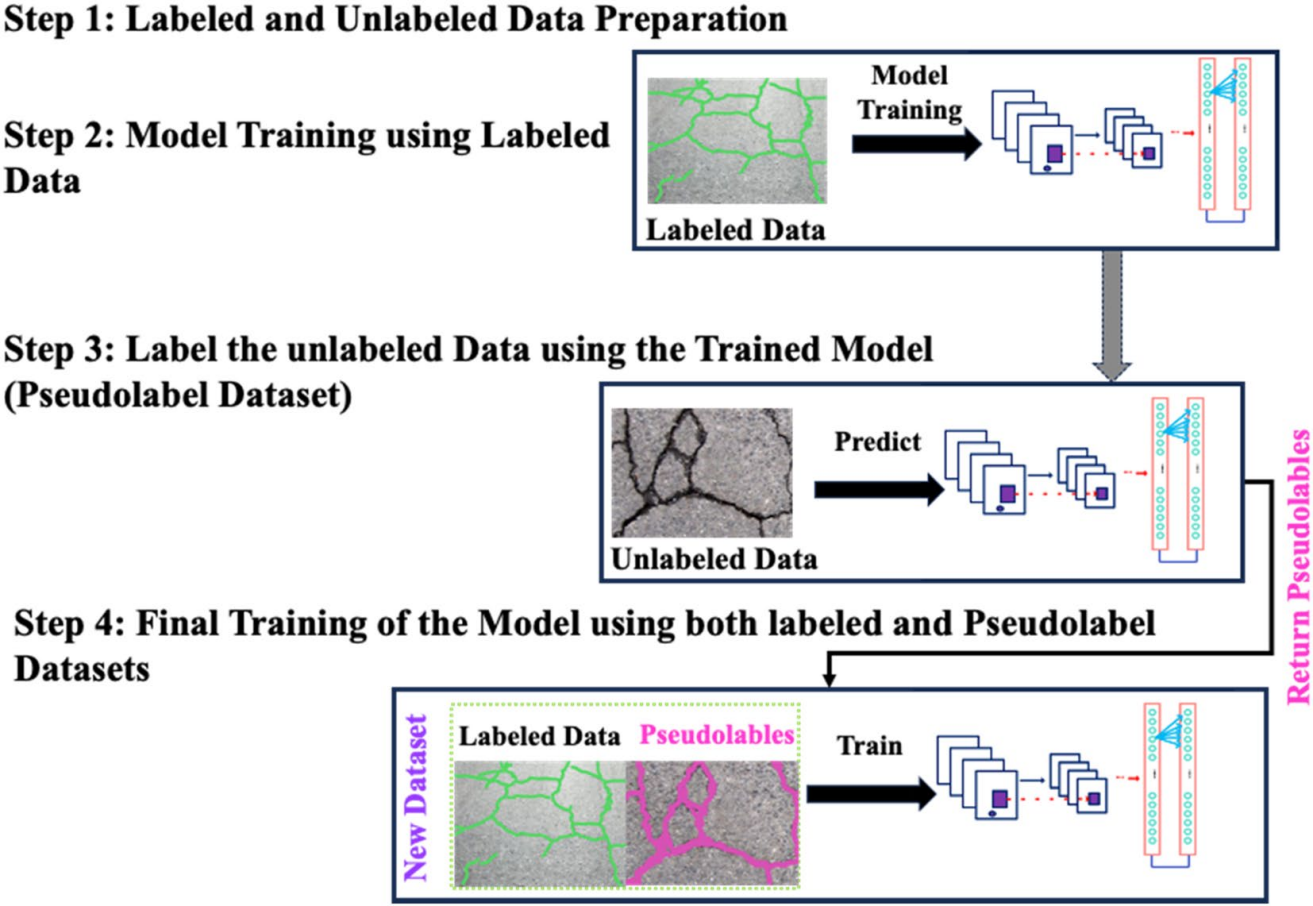
The depiction of traditional pseudo-labelling operation.

**Figure 5:**
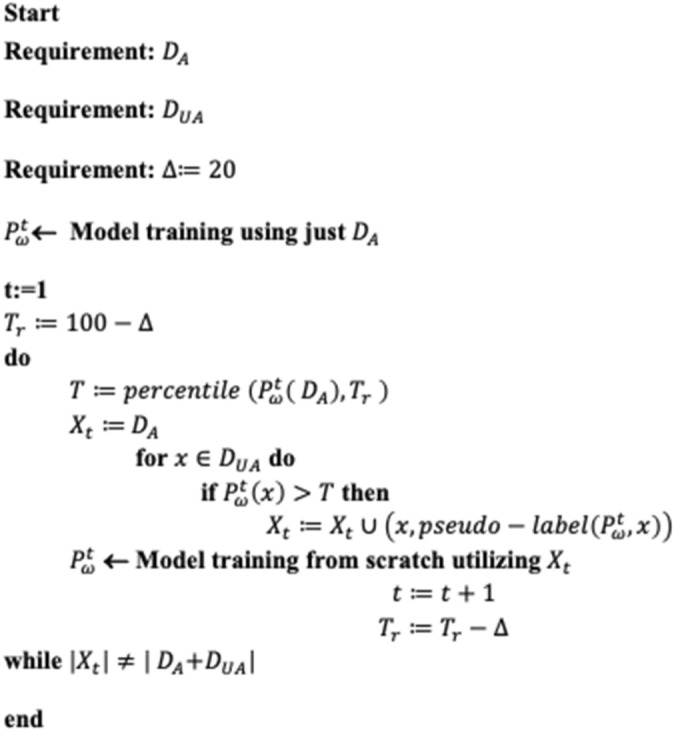
Curriculum learning algorithm for pseudo-labeling.

**Figure 6:**
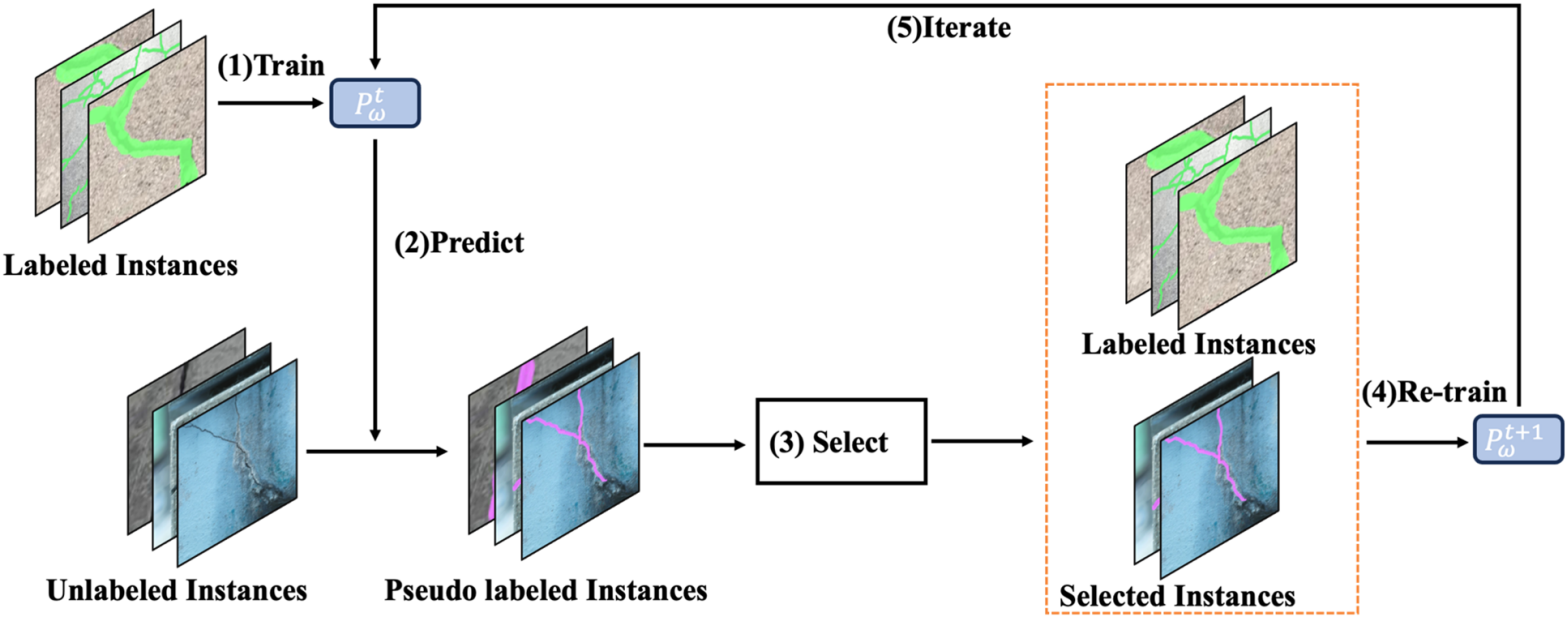
The Curriculum Labeling (CL) algorithm. Initially, the labeled instances are used to train the model, after which the trained model is utilized for predictions and the assignment of pseudo-labels to the unlabeled samples. Subsequently, a subset of pseudo-labeled instances is selected based on the distribution of the prediction scores. Following this, a new model is re-trained using the newly created dataset of labeled and pseudo-labeled instances. This process of relabeling unlabeled instances is iterated until all the instances in the datasets have been utilized.

In this study self-supervised pseudo-labeling approach is utilized due to the access to a limited-size training dataset as it is the case with crack detection and segmentation datasets. The process of standard pseudo-labeling approach is demonstrated in Fig. 4. The pseudo-labeling is semi-supervised process in which a model is trained on a combination of annotated and un-annotated data^50^. In semi-supervised learning, an annotated dataset ${D}_{A}=\left\{\left(i,l\right)|i\in \text{I}, l\in \text{L}\right\}$ and un annotated dataset ${D}_{UA}=\left\{i|i\in \text{I}\right\}$ are used, where $i$ denotes the inputs and $l$ denotes the labels. Typically, ${D}_{A}$ is much less than ${D}_{UA}$, i.e., $\left|{D}_{A}\right|\ll \left|{D}_{UA}\right|$. Pseudo-labeling approach is based upon the general principle of self-training^51^, where a model iteratively train itself by leveraging its previous predictions. Initially, model utilizes annotated data ${D}_{A}$ for its training, whereas, subsequently, uses ${D}_{A}$ and a pseudo-labeled subset of ${D}_{UA}$ in the previous cycle. In the standard pseudo-labeling approach, the results are prone to data distribution and conformation bias^52^. To mitigate these issues, a curriculum learning based self-supervised pseudo-labelling (CL-SSPL) technique introduced in Ref.^53^ is employed in this study. The pseudo-code of this self-supervised pseudo-labeling techniques is given below in Figs. 5 and 6 depicts the process of this enhanced self-supervised pseudo-labelling technique. This technique is based on the following two adjustments in the basic pseudo-labeling approach: (1) employing the principle of curriculum learning (CL), and (2) mitigating concept drift by resetting the parameters of the model at the inception of each cycle.

YOLOv8 and CL-SSPL based YOLOv8 employ different training approaches and methods for utilizing unlabeled data. The detection performance of YOLOv8 may be constrained by its reliance on manually labeled data, which may not generalize well to diverse or unseen scenarios. In contrast, the CL-SSPL based YOLOv8 model adopts a curriculum learning approach, gradually introducing unlabeled data with varying difficulty levels during training. This allows the model to learn from both labeled and unlabeled data, enhancing its adaptability and ability to generalize to various obscure and challenging conditions. By integrating curriculum learning and pseudo-labeling techniques, the CL-SSPL based YOLOv8, demonstrates improved robustness and performance, particularly in scenarios with limited availability of labeled data.

### CBAM attention mechanism

The backbone network incorporates convolutional block attention module (CBAM) at the end of the module to evade loss of the vital subtle information and filter output features maps produced by the hierarchical deep network. Unlike efficient Channel attention (ECA) and excitation (SE) modules, CBAM can simultaneously screening channel as well as spatial features. IN CBAM, weights are assigned to an input feature map via channel attention and spatial attention processes.

**Figure 7:**
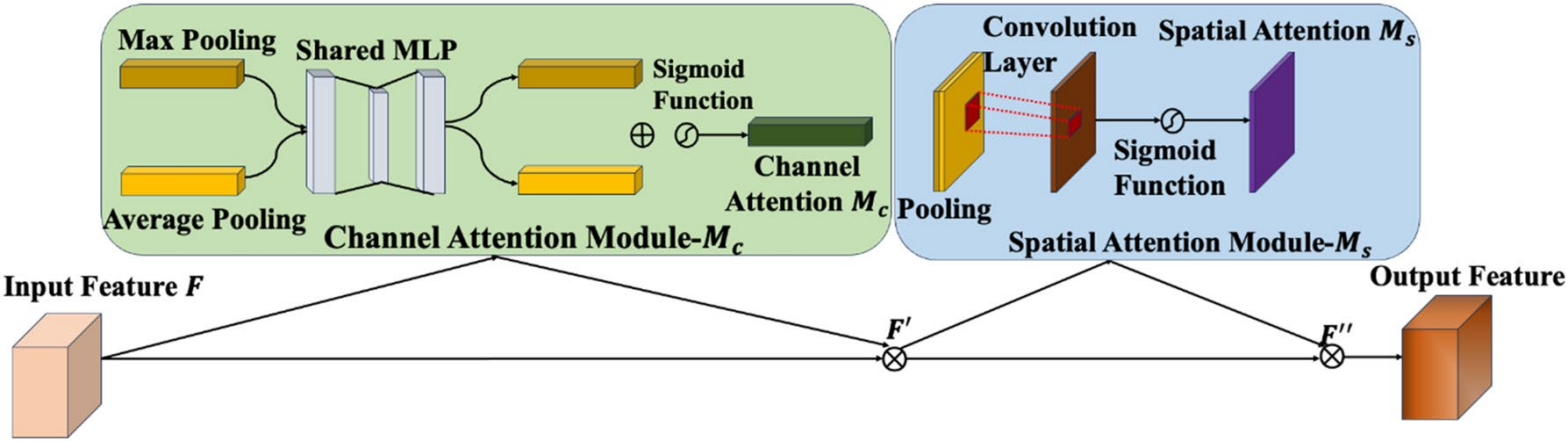
The representation of convolution block attention module.

The illustration of a CBAM attention module, shown in Fig. 7, is applied after the backbone feature extraction network. It weights the input feature map $F$, through combined channel and spatial attention operations. The weights are multiplied with the feature map to acquire the specific weight adjusted feature map. It helps in exploring the vibrant characteristics of fine-grained cracks. This approach has an advantage over other attention mechanisms, contributing to improved abstract feature exploration and discrimination capabilities within the SS-YOLO network.

### Gaussian adaptive weight distribution multi-head self-attention module

**Figure 8:**
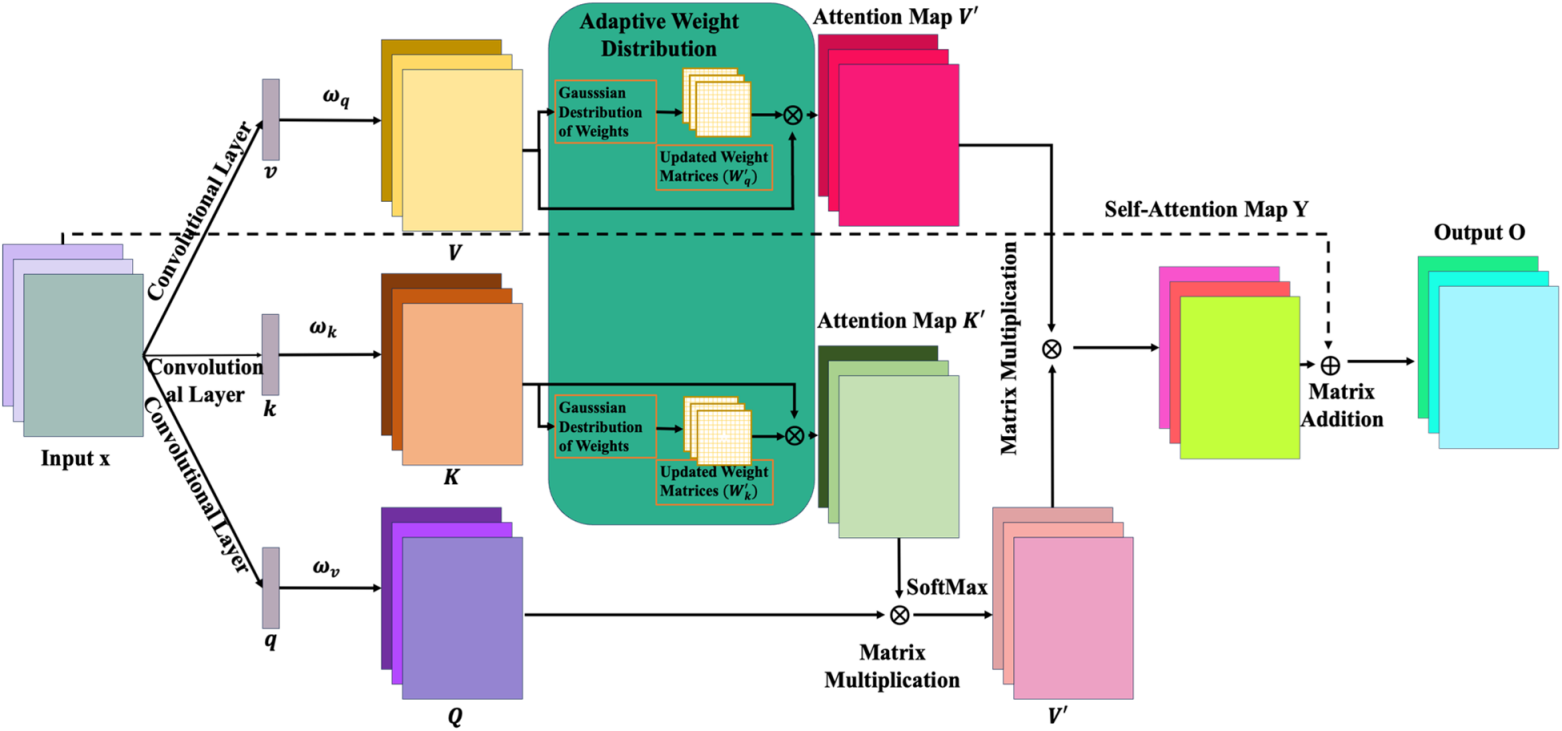
The architecture of adaptive weight mixing multi-head self-attention module.

Addressing the complexity of fine-grained cracks in diverse intricate background images, a Gaussian adaptive weight distribution multi-head self-attention module (GAWD-MHSA) given in Fig. 8 is incorporated in the Neck module of the SS-YOLO. The fine-grained cracks vary significantly in scale, aspect ratio, distribution pattern, and appearance. These variations portray a challenge for traditional YOLOv8 to effectually identify fine-grained cracks. The multi-head self-attention mechanism helps the SS-YOLO to specialize in capturing subtle details regarding crack and background, making crack detection process more effective. The integration of the GAWD-MHSA augments the ability of the model in combining and weighing different features during training. Especially, the Gaussian adaptive weight distribution (GAWD) mechanism enables it to collectively model probability distribution for dynamic recalibration of feature significance. This adaptive multi-head attention approach empowers the model in distinguishing fine-grained cracks from background pixels, expediting convergence.

The local feature map which is denoted by x is used as an input. Moreover, the three 1 × 1 convolution operations on x generate three feature vectors shown as $q$, $k$, and $v$ in the figure. These vectors contain positional information of each input feature vector. Therefore, these vectors are beneficial in strengthening the perception of sequence structure of the model. Next, expressions in Eq. (2) are used to formulate the correlations denoted by $Q$, $K$, and $V$. These are the correlations between corresponding local area features and global features.

2 $$\left\{\begin{array}{c}Q={\omega }^{q}q\\ K={\omega }^{k}k\\ V={\omega }^{v}v\end{array}\right.$$

During feature extraction GAWD alleviates the imbalance of local and semantic information. The GAWD computes the attention weights through Gaussian probability density function (GPDF). In GPDF, the mean, $\mu$, is adjusted by an offset, $\Delta$, and scaled variance, $\xi$, is a parameter which is learned. For each feature vector the mean “$\mu$” and the variance “${\sigma }^{2}$” is computed using the following expressions.

3 $$\mu =\frac{1}{T}\sum_{i=1}^{T}{x}_{i}, {\sigma }^{2}=\frac{1}{T}{\sum }_{i=1}^{T}{x}_{i}^{2}- {(\mu )}^{2}$$

The $\mu$ is adjusted using an offset, o, to derive $\varphi$ shown in Eq. (4). This helps the attention module to dynamically adjust the focus according to the distribution of input data. The aim is to compute the population mean not necessarily reflecting the characteristics of the current inputs. A vital normalization process is conducted on the input feature vector $x$ using adjusted mean, $\varphi$, as given in Eq. (5), where $\lambda >0$ for stability. This process is essential to stabilize the learning process and enhance the performance of the model.

4 $$\varphi =\mu +o$$

5 $${x}_{norm}=\frac{x-\varphi }{\sqrt{{\sigma }^{2}+\lambda }}$$

To compute the attention weights, the Gaussian function is applied to the norm and learnable scaled variance for each input vector. The computation of the attention weights through the application of Gaussian function is given in the following expression.

6 $$\text{GAWD}\left({x}_{i}\right)=\text{exp}\left(-\frac{{x}_{norm}^{2}}{2\varphi }\right)$$

The output feature map of self-attention mechanism is acquired through a series of equations, leading to the final output as shown in Eq. (9).

7 $${s}_{ij}=\text{softmax}\left(\frac{Q.{K}_{i}{\prime}}{\sqrt{{d}_{k}}}\right)$$

8 $$\text{y}={\sum }_{j}^{n}{s}_{ij}{V}{\prime}$$

9 $$\text{O}=\upomega {y}_{i}+{x}_{i}$$

Integrating CL-SSPL with CBAM and GAWD-MHSA into YOLOv8 yields several practical benefits. Firstly, the curriculum learning approach incorporates unlabeled data of increasing complexity levels, enabling the model to sequentially understand and learn more complex features, thereby enhancing its adaptability and generalization in various real-world scenarios. Secondly, CBAM, employing joint spatial and channel attention mechanisms, improves its ability to focus on region of images with key feature information. Thirdly, the fusion of GAWD-MHSA enhances feature representability through adaptive attention mechanisms, enabling the model to focus on relevant information while suppressing noise, resulting in improved detection precision, especially in complex or ambiguous environments. This integration enhances YOLOv8 with better performance, making it more robust, scalable, and applicable for detecting objects across a variety of tasks.

## Experimental setup

### Experimental setup and data description

For methodological transparency and contextual understanding, the experimental setup and datasets used in this research work are described in the subsequent sections. Familiarity with the experimental setup and data is also beneficial for reproducing the experiment and making comparison with previous work.

#### Experimental setup

The experimental hardware configuration included an Apple chipset featuring 12 cores for general processing and an additional 18 cores dedicated to graphics processing, equipped with 36 GB of video memory. The software environment utilized the macOS operating system version 14.1.2 and PyCharm 2023.3.

#### Evaluation metrics

The proposed SS-YOLO approach uses F1 score, recall, precision, mean average precision (mAP-0.5), Inference threshold in terms of intersection over union (IoU) and inference speed as evaluation metrices. The formula for the calculation of F1 score is stated below.

10 $$F1\_score= \frac{2(Precisoin\times Recall)}{Precisoin+Recall}$$

Additionally, Eq. (11) demonstrates the formulation of mean average precision ($mAP$) calculation. It is the mean of the average precision of the instances in all classes and is used to evaluate the crack detection and segmentation performance of the model.

11 $$mAP=\frac{1}{M}\sum_{i=1}^{T}{AP}_{i}$$

Furthermore, the IoU is calculated by dividing the area of overlap (AoO) between predicted and actual bounding boxes with area of union (AoU) between the predicted and actual bounding boxes. To calculate the IoU the following expression can be utilized.

12 $$IoU=\frac{AoO\;between\;the\;bounding\;boxes}{AoU\;between\;the\;bounding\;boxes}$$

#### Dataset description

**Figure 9:**
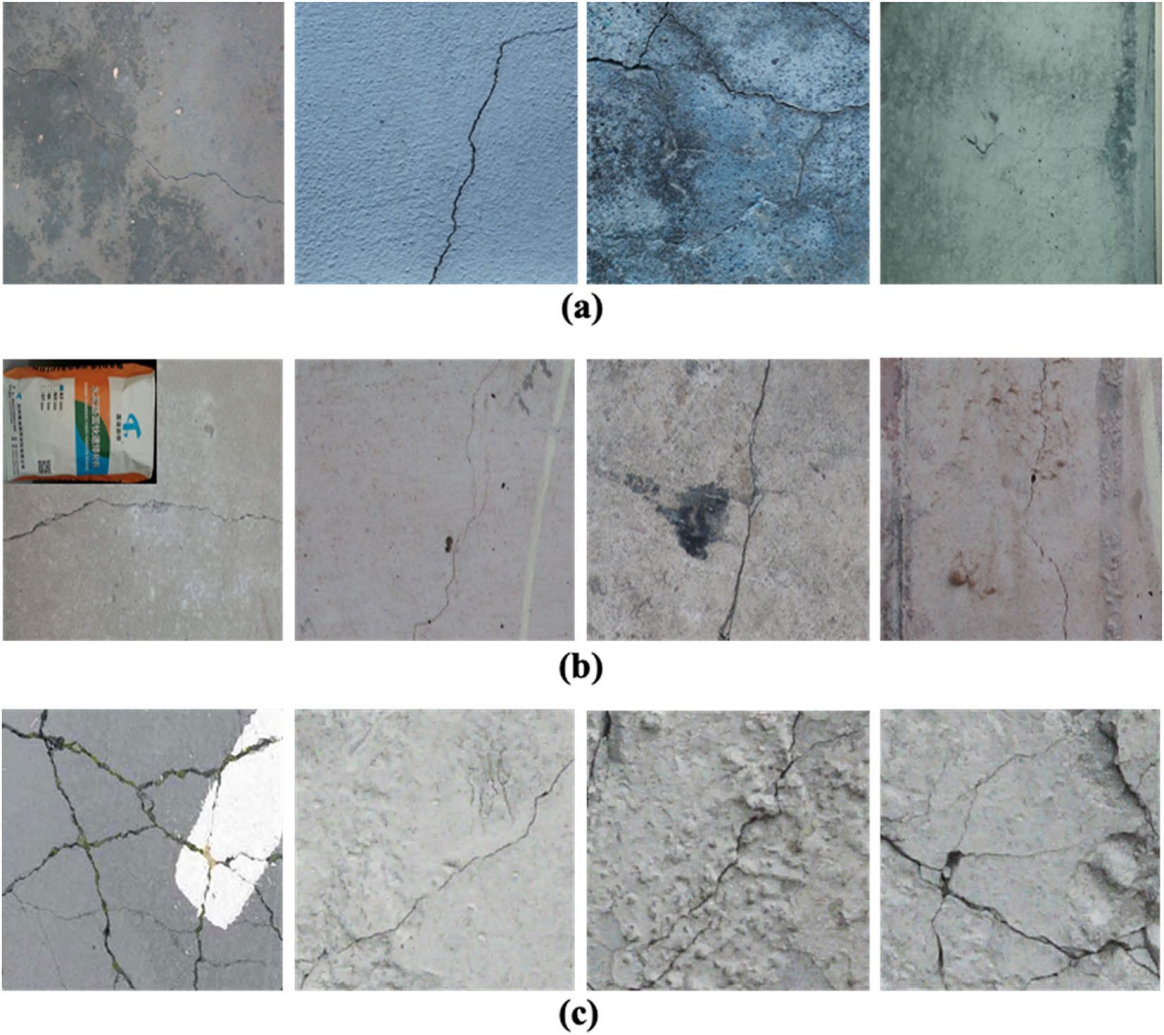
A few images from the three datasets (**a)** Crack-Detection-and-Segmentation Dataset, (**b**) DeepCrack Dataset, and (**c**) FCN-Crack Dataset.

To device and weigh the efficacy and generalization capacity of the proposed model, three distinct datasets containing surface cracks in concrete structures are used in this study. Multiple datasets are considered because the publicly available datasets typically consist of limited-size data. Samples from a public dataset^54^ are used to train the proposed model. The other two datasets, i.e., DeepCrack^55^, and FCN-Crack^56^ datasets are used to check the generalizability of the proposed SS-YOLO model. Figure 9 presents exemplary samples from the three datasets. Apart from their small size, fine-grained cracks possess narrow and complex shapes, resulting in a cluttered appearance. As evidenced by samples from various datasets, the images contain fine-grained cracks that are minute in nature, exhibiting complex skeletons and cluttered views. Additionally, these datasets include macro cracks with irregular patterns and complex skeletons. The irregular and complex nature of these skeletons poses a challenge for crack detection. Further details regarding these datasets are presented in the subsequent sections. Apart from their small size, fine-grained cracks possess narrow and complex shapes, resulting in a cluttered appearance. As evidenced by samples from various datasets, the images contain fine-grained cracks that are minute in nature, exhibiting complex skeletons and cluttered views. Additionally, a few samples of these datasets contain macro cracks with irregular patterns and complex skeletons. The irregular and complex nature of these skeletons poses a challenge for crack detection. Further details regarding these datasets are presented in the subsequent sections.

##### Crack detection and segmentation dataset

A comprehensive dataset was compiled in^54^, containing images with a resolution of 450 × 450 pixels. It consists of images with cracks in a variety of concrete structures suitable for crack detection and segmentation task. This diverse dataset includes images from various concrete structures such as roads, bridges, and buildings. After preprocessing, 4215 images containing labels suitable for the crack segmentation process were utilized in this research. The database serves as both the training set and one of the validation datasets to assess the efficacy of the proposed SS-YOLO network for crack detection and segmentation in concrete structures. During the training phase, 75% of the instances were used, while the remaining 25% were allocated for model validation. The arrangement of the images in the dataset is detailed in Table 1.

**Table 1:** The split of the dataset in training, and validation subsets.

Total no. of images in the dataset	Details of the images in the subsets	
	Training (75%)	Test (25%)
4215	3161	1054

##### Deepcrack dataset

The dataset given in Ref.^55^ comprises 537 original color images and corresponding manually annotated segmentation labels. Each segmentation label is represented by a binary image representing pixel-wise segmentation mask, precisely representing the crack regions. All images share a fixed size of 544 × 384 pixels and were used to test the generalization capability of the proposed model.

##### FCN-crack dataset

A challenging publicly available dataset^56^ is also used in this study to check the generalizability of the proposed model to discern shapes at the image level. It amasses over 800 images containing crack widths from one pixel to 100 pixels. To ensure diversity, these images contain pavement cracks as well as cracks on concrete walls. The images are captured at varying distances corresponding to their sizes, resulting in resolutions spanning from 72 to 300 dpi. The ground truth data is obtained by manually annotating the images at the pixel level. The examiners annotated background pixels as zero, while crack pixels as one.

### Declaration of generative AI and AI-assisted technologies in the writing process

During the preparation of this work, the author(s) used Grammarly in order to improve the manuscript grammatically. After using this tool/service, the author(s) reviewed and edited the content as needed and take(s) full responsibility for the content of the publication.

## Results and analysis

### Model parameter optimization

**Figure 10:**
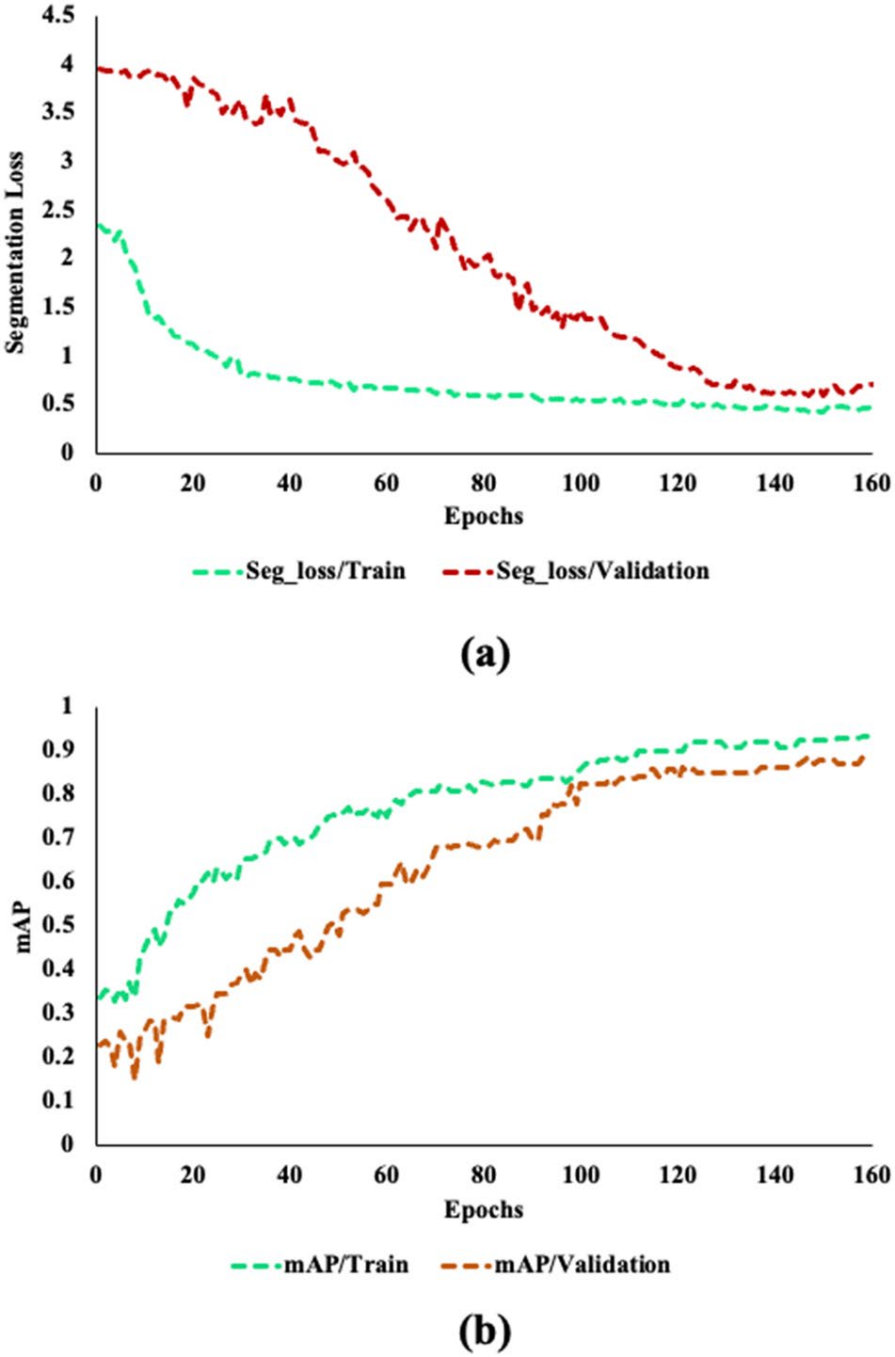
The plots for the segmentation loss and mean average precision (mAP) during training and validation phases, (**a**) segmentation loss for training and validation phases, (**b**) mAP for training and validation phases.

The SS-YOLO model underwent training and testing on a crack image dataset, with a batch size set to 16, an initial learning rate of 0.0001, and 150 epochs. The AdaMax optimizer was used for weight optimization, employing the maximum norm of gradients for dynamic adjustment of the learning rate to achieve better convergence. To diversify the data samples, during training of the model, data augmentation procedures including, samples flipping, considering various rotations, scaling are considered. Model evaluation, as depicted by the smoothed curves in Fig. 10, evidently shows a consistent decay in total losses during training (Fig. 10a) and an enhanced mean Average Precision at 0.5 (mAP) of 91.5% (Fig. 10b). The SS-YOLO model demonstrated faster convergence and higher accuracy compared to the original YOLOv8, surpassing it by 5.1 percentage points.

### Result analysis

This study employs a self-supervised network, namely SS-YOLO, for the detection and segmentation of fine-grained cracks using limited-size data. To infer cracks on unseen data, an intersection over union (IoU) threshold of 0.85 was used. Moreover, to achieve better detection and segmentation results image level data augmentation methods such as, varied orientation, rescaling, variance in contrast and noise levels were applied to develop the model. Performance metrics, including, precision, recall, mAP, IoU threshold, and inference speed are presented in Table 2 for the SS-YOLO model during both training and validation stages. During the training phase it is evident that the highest mAP and F1 score achieved are 91.5% and 0.88%, respectively. The proposed SS-YOLO model also has the highest precision and recall as compere to the other models. For the traditional YOLOv8s, the performance metrics are 88.24% mAP and 0.83% F1. The lowest performance is observed for YOLOv5s, with mAP and F1 of 85.6% and 85%, respectively.

**Table 2:** The details of the comparison for various YOLO models in terms of different performance metrices.

Model	Train				Validation					Inference Time (milliseconds per image)
	mAP (%)	Precision (%)	Recall (%)	F1 score (%)	IoU threshold	mAP (%)	Precision (%)	Recall (%)	F1 score (%)	
YOLOv5s	85.60	92.5	81.2	85.00	0.50	85.10	91.56	77.11	83.72	10
YOLOv6s	85.90	92	80.21	85.70	0.50	89.50	89.91	74.36	81.40	13
YOLOv8s	88.24	91.00	77.00	83.00	0.50	86.50	89.91	78.00	82.00	12
SS-YOLO	91.50	93	83.71	88.11	0.85	90.80	91.14	83.25	87.01	18

In the validation stage, a similar trend can be observed in the performance of the models. The SS-YOLO model demonstrates the highest precision and recall values, followed by traditional YOLOv8 and YOLOv5. Additionally, the proposed model exhibits slightly longer inference times for detecting cracks in single image compared to the other algorithms. However, the difference in inference times is marginal and negligible. These optimal evaluation metrics in both phases indicate the readiness of the model for detecting cracks. It is worth noting that the proposed model yielded superior inference results using a comparatively higher Intersection over Union (IoU) threshold of 0.85.

**Figure 11:**
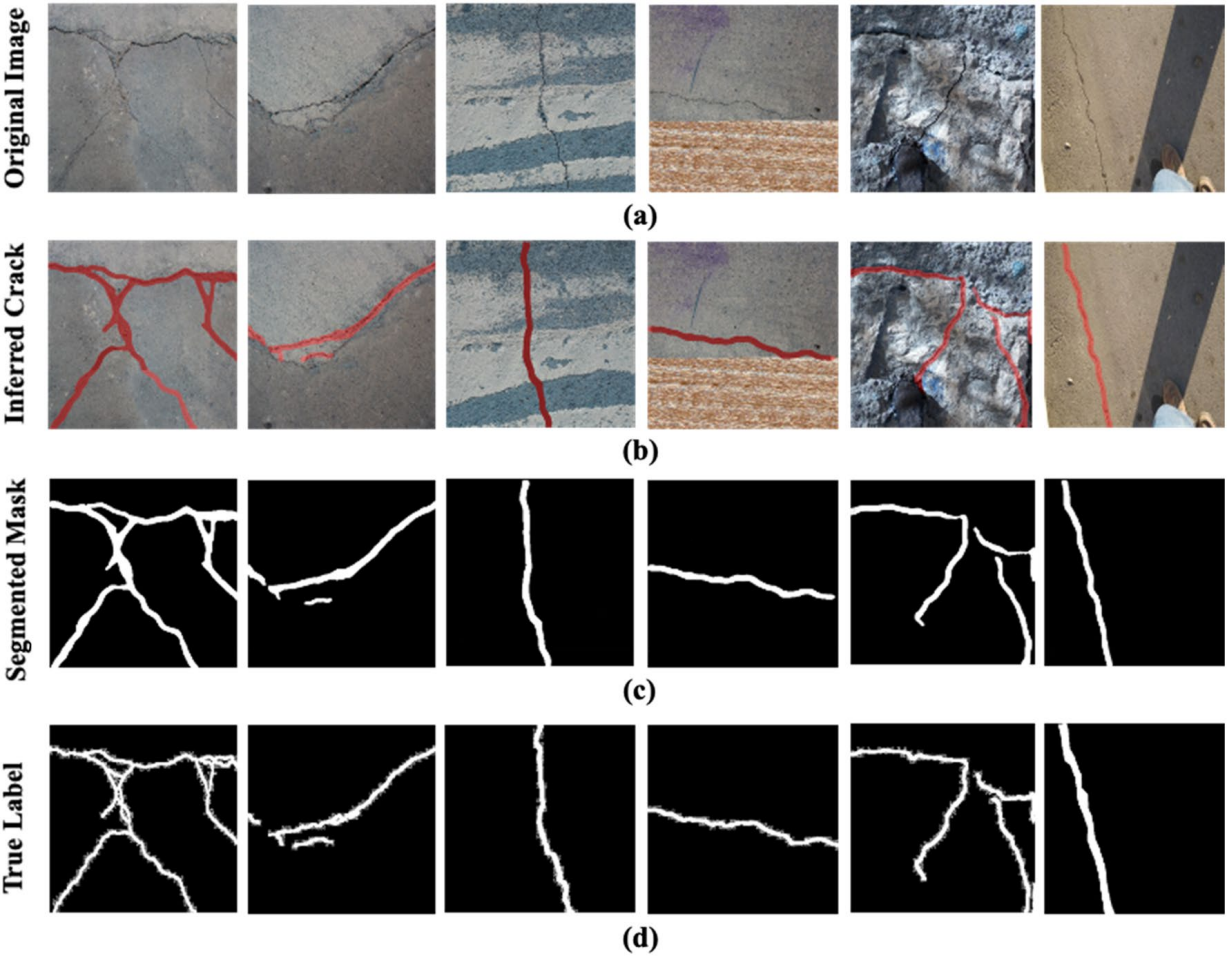
The Inference and segmentation of cracks through the proposed SS-YOLO model. (**a**) original images, (**b**) inferred cracks via SS-YOLO model, (**c**) Segmented masks of the inferred cracks, (**d**) the true labels of the images.

As discussed in “Experimental setup”, the proposed model is assessed on the test subset comprising 1578 images. In Fig. 11a–d depict the original images, inference results, segmented masks of the inferred cracks, and true labels of the images. The sample images presented in Fig. 11a contain images with cracks from diverse concrete structures. Notably, the varying texture and illumination conditions increases the intricacies of the background. Nevertheless, as evident in Fig. 11b, the proposed model inferred cracks in the unseen images with intricate backgrounds with high confidence. As a result, there is a high resemblance in the segmented masks given in Fig. 11c and true labels shown in Fig. 11d.

### Performance comparison of different detection networks

**Figure 12:**
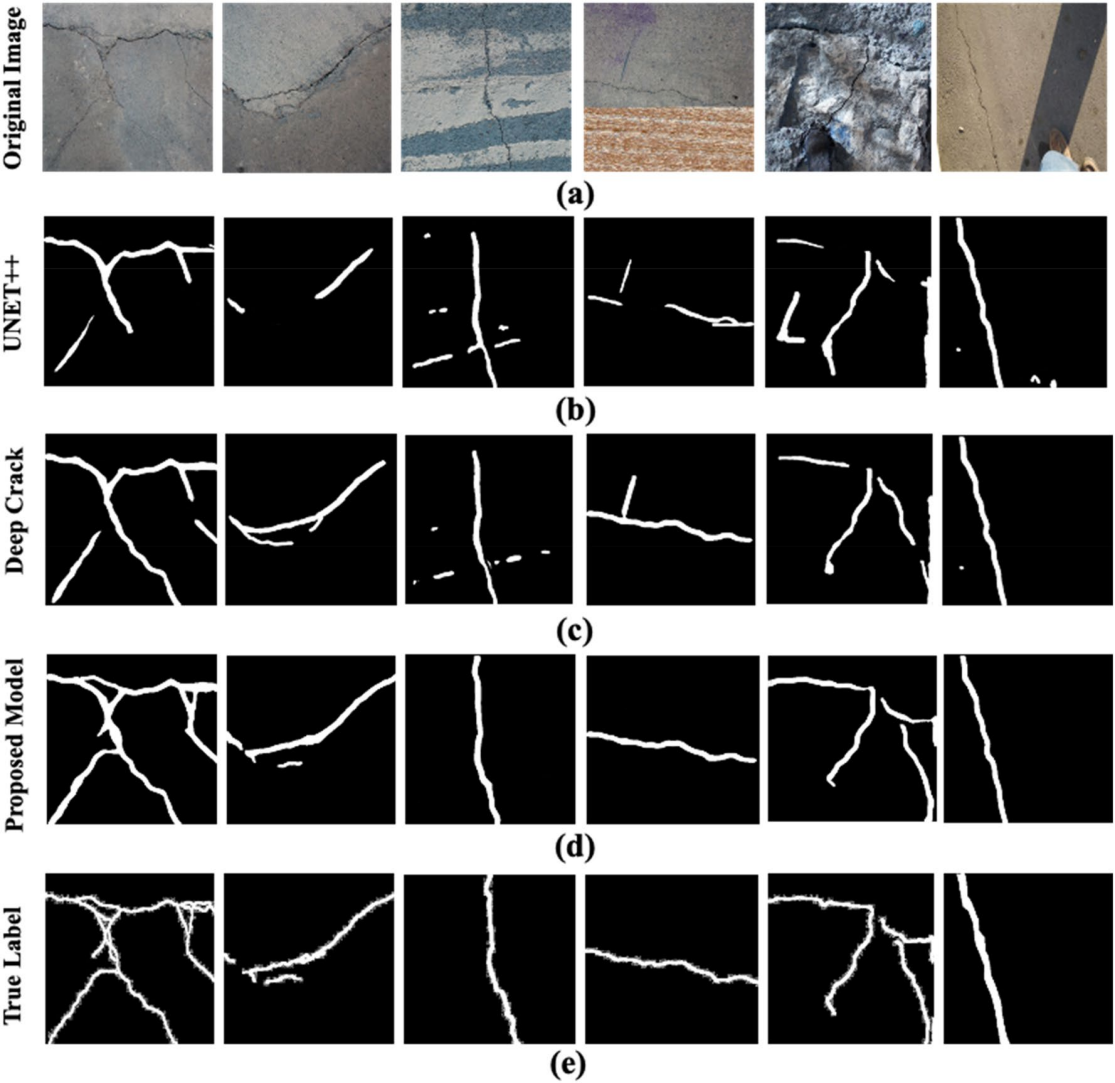
The comparison results of the SS-YOLO model with other state of the art crack detection models: (**a**) original images, (**b**) Segmented masks of the inferred cracks through UNET++, (**c**) Segmented masks of the inferred cracks through DeepCrack, (**d**) Segmented masks of the inferred cracks through the proposed SS-YOLO model, (**e**) the true labels of the images.

Figure 12a–e illustrate original images and segmented masks produced on the test dataset by different models alongside with the true labels. The images in Fig. 12a depict intricate backgrounds and diverse lighting conditions, emphasizing the challenge of the segmentation task. Notably, in Fig. 12d, the segmentation masks produced by our proposed approach bear a resemblance to the true labels depicted in Fig. 12e. This semblance is despite the presence of cracks in images with intricate backgrounds and diverse lighting conditions as shown in Fig. 12a.

Furthermore, the segmentation results of the proposed model are also compared with those of state-of-the-art (SOTA) crack detection models. These SOTA models specify the advanced version of the deep U-shaped Network (U-NET), known as U-NET++, and DeepCrack. These comparisons are showcased in Fig. 12c,d. It is evident from the images that the segmentation performance of U-NET++ and DeepCrack visibly diminishes in the presence of challenging backgrounds and lighting conditions. The segmentation outcomes given in Fig. 12 substantiate the preeminence of the proposed SS-YOLO model in accurately segmenting cracks.

### Generalizability validation

To validate the generalization power of the proposed model, it was tested on the DeepCrack^55^ and FCN-Crack datasets^56^. The DeepCrack dataset comprises 537 images of concrete structures with a resolution of 544 × 384, while the FCN-Crack dataset contains 800 images with resolutions ranging from 72 to 300 dpi.

**Figure 13:**
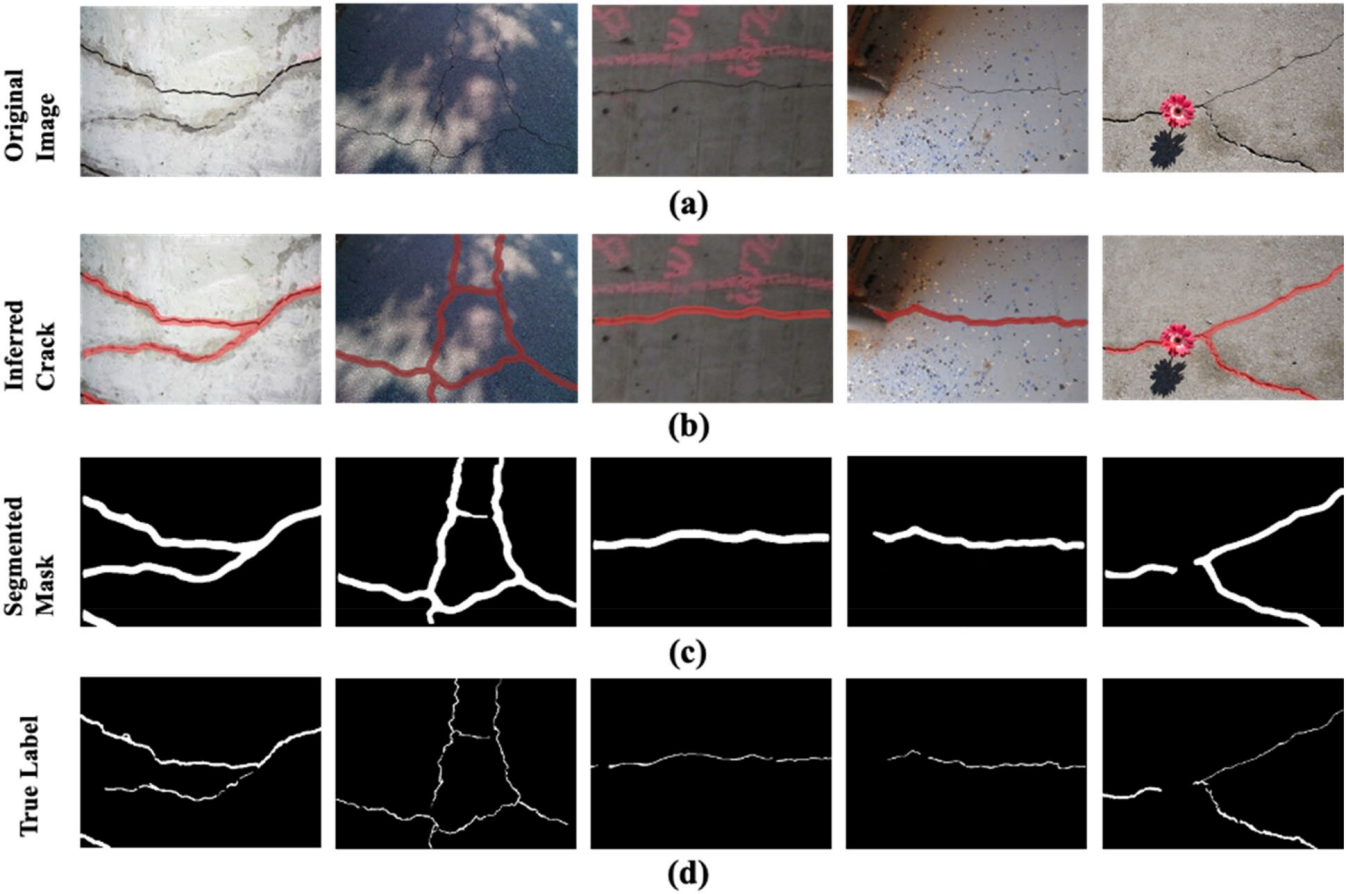
The results of the proposed SS-YOLO model on deep crack dataset: (**a**) the original images, (**b**) the inferred cracks via SS-YOLO model, (**c**) Segmented masks of the inferred cracks, (**d**) the true labels of the images.

Detecting and segmenting fine-grained cracks from the DeepCrack and FCN-Crack datasets pose additional challenges due to the presence of multi-scale and multi-scene images. These images also contain noise from stains, spots, undesired objects, and uneven illumination, increasing the complexity of crack identification, particularly in ground regions. The irregular and asymmetrical patterns of cracks, such as diagonal and crazing patterns, further complicate the segmentation process. Figure 13b,c demonstrate the commendable ability of the proposed model to infer and segment cracks using DeepCrack dataset. The results depict the segmentation efficacy of the model on cracks with complex skeleton and distribution tessellations. Furthermore, as shown in Fig. 13c, the segmented masks closely resemble the true labels in Fig. 13d, affirming the effectiveness of our proposed model. These outcomes support the notion that our model exhibits strong generalization capabilities, successfully inferring cracks in images with varying scales, illuminations, noise, and intricate backgrounds.

**Figure 14:**
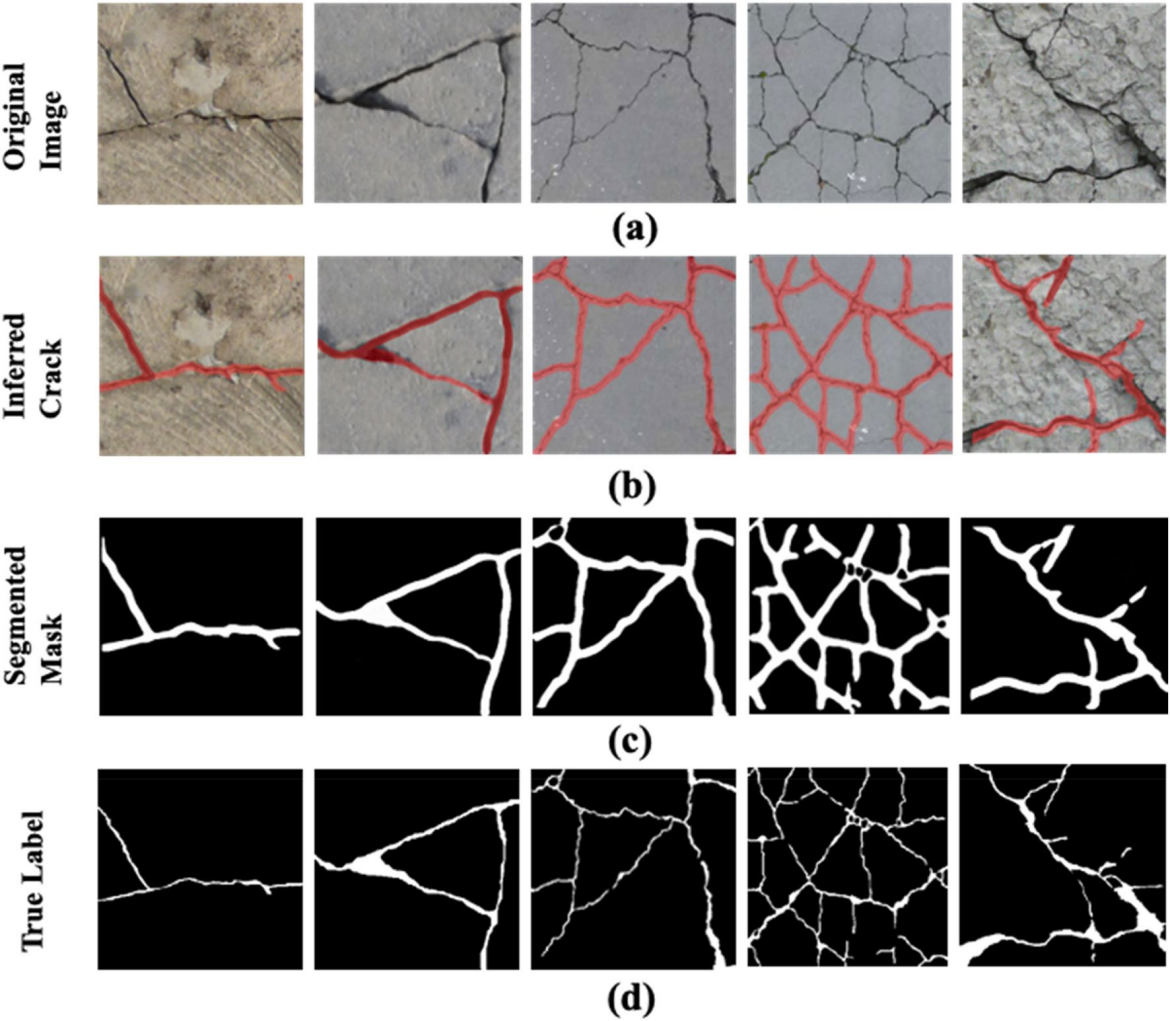
The results of the proposed SS-YOLO model on FCN-crack dataset: (**a**) the original images, (**b**) the inferred cracks via SS-YOLO model, (**c**) Segmented masks of the inferred cracks, (**d**) the true labels of the images.

Furthermore, the qualitative results of SS-YOLO model for segmenting images in FCN-Crack dataset are presented in Fig. 14a–c. In Fig. 14b,c, it is visible that our SS-YOLO model accurately identifies cracks in complex images but occasionally includes outliers like intersection points due to the complexity added by crazing cracks. Nonetheless, the overall segmentation results thoroughly align with the true labels provided in Fig. 14c.

In addition to the qualitative results, the proposed model is evaluated using different training and testing configurations as outlined in Table 3. It is evident that the proposed model achieves its highest performance when trained with the crack detection and segmentation dataset and tested with the remaining datasets. This can be attributed to the diversity and size of the training dataset. During the training phase, the network effectively explores diverse and distinct information related to crack detection and segmentation processes. Conversely, the network exhibits its lowest performance when trained on the DeepCrack dataset and tested on the other datasets. This decline is attributed to the limited size of the dataset. It can be inferred that training on big data containing a large number of samples is beneficial for training deep networks, as they can effectively explore distinctive information from the input data. To further consolidate the generalizability of the proposed model, we conduct a comprehensive examination of mAP, F1 scores, and inference speed and compared to prevailing SOTA techniques in Table 4. These metrics are crucial for estimating the performance of the model and were computed using three distinct datasets: the crack detection and segmentation dataset^54^, the DeepCrack dataset^55^, and the FCN-Crack dataset^56^.

**Table 3:** The details of the evaluation metrics for the proposed SS-YOLO model under different training and testing configurations.

Train dataset	Inference threshold (%)	Test dataset	Precision (%)	Recall (%)	F1-score (%)
Crack detection and segmentation dataset	85%	Deep crack	90.53	83.77	87.01
		FCN-crack	89.91	82.81	86.21
Deep crack dataset		Crack detection and segmentation dataset	87.42	79.18	83.09
		FCN-crack	87.63	80.04	83.66
FCN-crack dataset		Crack detection and segmentation dataset	88.79	81.83	85.16
		Deep crack	88.86	82.04	85.48

**Table 4:** The details of the evaluation metrics for the proposed model and other state-of-the-art models.

Method	mAP (%)	F1 Score (%)	Inference time (milliseconds per image)
Crack detection and segmentation dataset			
U-NET	82.50	83.12	29.00
U-NET++	83.43	86.00	30.00
YOLOv5-MobileNetv3^57^	87.10	84.00	15.19
EfficientDet^58^	50.00	46.00	37.03
Proposed	90.80	88.00	18
Deepcrack dataset			
U-NET	81.96	80.88	27.00
U-NET++	82.67	82.82	28.00
YOLOv5&MobileNetv3^57^	87.24	84.56	15.00
EfficientDet^58^	51.07	46.15	35.01
Proposed	89.93	88.93	18
FCN-Crack dataset			
U-NET	82.00	80.10	23.68
U-NET++	83.63	81.93	25.01
MN-YOLOv5^57^	87.00	83.99	14.73
EfficientDet^58^	49.90	46.00	34.96
Proposed	89.62	88.76	17.42

For the Crack Detection and Segmentation Dataset, our proposed model consistently outperforms all the other SOTA models across all evaluation metrics, demonstrating noteworthy improvements. Specifically, we observe a minimum improvement of 4.40% and 2% in mAP and F1 values, respectively, compared to the models. Notably, our model achieves higher mAP while maintaining a shorter inference time, approximately 11 ms per image less than the comparative models, except MN-YOLOv5^57^. Inference time is a critical consideration, especially in scenarios requiring rapid decision-making or administering large image volumes in real-time.

Similarly, in the DeepCrack and FCN-Crack datasets, the proposed SS-YOLO model demonstrates superior performance. It achieves the highest mAP and F1 scores while maintaining a faster inference speed. Compared to other networks on both datasets, the proposed SS-YOLO model shows a minimum mAP improvement of 2.62%. Additionally, enhancement of at least 6.83% was observed in F1 score. Moreover, the inference time taken per image by the proposed SS-YOLO is model at least 2 ms less on both datasets. The outperformance of the proposed SS-YOLO model across different evaluation metrics highlights its crack inference capability using diverse datasets, substantiating its generalization ability.

### Ablation analysis

Table 5 highlights the performance of the model under different configurations. It is evident that the base model, without any architectural or training process augmentation, is the least efficient. In this configuration, the highest values for mAP, F1 score, and inference time are 86.16%, 81.34, and 12 ms, respectively. Furthermore, when data augmentation is employed during model training, there is a noticeable improvement in evaluation metrics. This improvement is evidenced by a 2.08% increase in mAP value and a 1.66% increase in the F1 score.

**Table 5:** The effect of the CL-SSPL and the attention modules on performance metrics.

Ablation setting	mAP (%)	F1 score (%)	Inference time (millisecond per image)
YOLOv8s (w/o Data Augmentation, w/o CL-SSPL and the Attention Modules)	86.16	81.34	12
YOLOv8s (with Data Augmentation, w/o CL-SSPL and the Attention Modules)	88.24	83.00	12
YOLOv8x (with Data Augmentation and CL-SSPL and w/o Attention Modules)	88.26	83.14	12
YOLOv8s (with Data Augmentation, CBAM Attention Module and w/o CL-SSPL)	88.85	83.13	15
YOLOv8s (with Data Augmentation, GAWD-MHSA Module and w/o CL-SSPL)	89.05	83.69	16
YOLOv8s (with Data Augmentation, both the Attention Modules and w/o CL-SSPL)	90.17	87	18
YOLOv8s (with Data Augmentation, CBAM Attention Module and CL-SSPL)	89.28	83.44	15
YOLOv8s (with Data Augmentation, GAWD-MHSA Module and CL-SSPL)	89.54	85.06	16
SS-YOLO	90.80	88	18

The significance of incorporating GAWD-MHSA and CBAM modules is also apparent from the table. The CBAM module, in conjunction with data augmentation, enhances the mAP value by 2.69% and the F1 score by 1.79% compared to the base model. Similarly, when comparing to the base model, the GAWD-MHSA module with data augmentation elevates the mAP and F1 values by 2.89% and 2.35%, respectively.

Moreover, the integration of both modules significantly improves the performance of the conventional YOLOv8s model. This improvement is substantiated by a 4.01% increase in mAP and a 5.66% increase in F1 values compared to the base model. These architectural updates, especially GAWD, collectively model the probability distribution for dynamic recalibration of feature significance, enhancing the model's ability to combine and weigh different features during training.

Additionally, CL-SSPL positively impacts the performance of the YOLOv8 model, as observed by a 2.08% increase in mAP and a 1.66% increase in the F1 score compared to the base model, considering the constraint of limited-size training data.

Moreover, the proposed SS-YOLO approach, which incorporates both attention mechanisms and CL-SSPL, demonstrates outstanding performance. The F1 score and mAP are boosted by 6.66% and 4.64%, respectively. The crack inference time of the proposed SS-YOLO model is slightly higher due to the incorporation of a higher number of additional modules compared to other configurations. However, the increase in inference time is negligible, as it is slightly higher than the base model. This trade-off in inference time is justified by the superior performance of the model in detection and segmentation of fine-grained cracks with high generalization power.

## Discussion

The ingress of cracks is considered an early sign of deterioration in a concrete structure. Identifying cracks in civil infrastructures is inevitable, as stability and resilience are compromised by their presence. Although numerous crack detection and segmentation techniques have been proposed over time, these techniques experience difficulties under challenging detection scenarios. The presence of written inscriptions, debris, extra objects, background shadows, and varying textures adds to the complexity of background pixels. In practice, it is cumbersome to detect and segment fine-grained cracks with state-of-the-art (SOTA) techniques in their standard form, as described in Fig. 12. The description is complemented by the results presented in Table 3. These results emphasize that architectural amendments are vital in precisely inferring fine-grained cracks in complex scenarios.

One architectural amendment to boost the detection accuracy of a network is to introduce a sophisticated attention mechanism. The attention mechanism emphasizes the foreground pixels, in this case, the pixels of fine-grained cracks during the detection process. Hence, it significantly improves the performance of a detection network. In this case, the integration of CBAM and GAWD-MHSA modules has assisted the model in exploring meaningful insights and variations associated with these cracks. These attention modules empower the proposed model to highlight pertinent details, making the model more resilient in coping with intricate backgrounds and salient information. The results presented in Figs. 11, 12, 13 and 14 and the ablation analysis performed in Table 4 support this statement.

Furthermore, the performance of a supervised model is compromised when trained on a limited-size dataset. In such cases, the model does not have exposure to diverse samples during the training process. As a result, due to the lack of diversity in the samples, the model learns limited information associated with a uniform set of samples. This obstruction can be avoided using the pseudo-labeling concept, where the model is iteratively trained on the predictions made by the model with high confidence. In this regard, the integration of CL-SSPL has a significant impact on the crack detection and segmentation performance of the model. This is justified by the results presented in Table 4 when the CL-SSPL module is incorporated for training the network.

Moreover, data augmentation such as flipping of samples, varying rotations, and scaling can also have a positive impact on the performance of the model. These operations adversely affect the training time taken by the model but introduce variations in the training samples. Therefore, through these variations, the learning ability of the model is significantly improved.

Although the proposed SS-YOLO model could introduce self-supervised learning ability by leveraging CL-SSPL, it still requires labeled data at the initial stage for training the network. The CL-SSPL technique is applied to diversify the dataset by using unlabeled data at a later stage for better training of the network. However, in practical scenarios, labels for a given dataset are often unknown. Under such circumstances, developing a base model using the proposed approach can be challenging. Nevertheless, the proposed approach has the potential to incorporate source-free unsupervised learning criteria into its framework.

## Conclusion

This work proposes a robust methodology based on self-supervised Yolov8 model referred to as SS-YOLO for inferring and segmenting fine-grained cracks using limited-size dataset containing images with complex backgrounds. The training process of the designed network is streamlined with a curriculum learning-based self-supervised pseudo-labeling (CL-SSPL) technique. The adaptation of CL-SSPL considerably augments the learning ability of the proposed model on limited-size datasets, mitigating the data imbalance issue and enhancing generalizability. It also regulates the training process and assist in adapting to varying distributions of the data. Additionally, attention mechanism is introduced in traditional YOLOv8 model in the form of Convolutional Block Attention Module (CBAM) and the Gaussian adaptive weight distribution multi-head self-attention module (GAWD-MHSA). The integration of these two attention modules supplements the ability of the YOLOv8 model to effectively capture subtle details associated with fine-grained cracks. Hence, the proposed model prioritizes relevant information through attention process.

The efficacy and generalizability of the proposed model is assessed on three distinct datasets. These datasets were enriched with images in diverse concrete structures such as buildings, pavements, and roadways. The images contained obstructions such as varying background shadows and texture, debris, and presence of unwanted objects that made the crack detection and segmentation process challenging. The primacy of the proposed model in terms evaluation metrics is evident from the experimental results. With the proposed model an increment of at least 2.89% and 4%, was observed in the mAP and F1 score. The ability of the proposed model to infer and segment fine-grained cracks in images with complex backgrounds is justifiable though enhanced segmentation results accompanied with ablation analysis. Moreover, the inference time per image of the proposed model is also at least 11 ms faster than other models in comparison, making it adaptable in practical scenarios.

In conclusion, primarily this paper contributes to the training process of a network by using the CL-SSPL technique as well as the inclusion of CBAM and MHSAM modules for exploring subtle details associated with fine-grained cracks amidst complex backgrounds. These amendments in the basic YOLOv8 model reduces the possibility of overfitting and enhances the detection performance of fine-grained crack with optimal inference time suitable for real-time applications.

## Data Availability

This work is conducted by using open-access dataset. The details are included in the dataset description section. Kindly refer to that and follow the cited references.
